# Risk assessment of agressive behavior in schizophrenia and schizoaffective disorder : a cross-sectional study

**DOI:** 10.1192/j.eurpsy.2023.2223

**Published:** 2023-07-19

**Authors:** A. Mellouli, N. Charfi, I. Gassara, N. Smaoui, S. Omri, R. Feki, L. Zouari, M. Maâlej, J. Ben Thabet, M. Maâlej Bouali

**Affiliations:** Psychiatry C Department, Hedi Chaker University Hospital, Sfax, Tunisia

## Abstract

**Introduction:**

Psychotic disorders have been consistently associated with aggressive behaviors. Psychiatrists are frequently asked to perform assessment regarding potentially aggressive patients. Thus, many psychometric instruments can be useful for identifying the risk of violence and thereby offering appropriate treatment for these individuals.

**Objectives:**

The aims of this study were to assess the risk of agressive behavior in inpatients with schizophrenia or schizoaffective disorder and to determine its correlates.

**Methods:**

Using face-to-face interviews, inpatients diagnosed with schizophrenia or schizoaffective disorder, in psychiatric department of the University Hospital in Sfax (Tunisia) were included in this cross-sectional, descriptive and analytical study, carried out between novembre 2020 and octobre 2022.

The modified overt aggression scale (MOAS) and historical clinical risk management-20 (HCR-20) questionnaire were used for data acquisition. The HCR-20 score of 20 was used as threshold to divide the sample to violent patients (scoring>20) and non-violent patients (scoring ≤ 20).

**Results:**

The sample consisted of 60 male inpatients. The mean age was 38.23± 10.37 years.

In our sample, 68.3% were single, 35% didn’t reach the secondary educational level, 16.7% used psychoactive substance(s), 35% had prior criminal record, 30% had a history of suicidal attempt and 81.7% had previous hospitalization.

The mean score of MOAS was 13.08±8.19. The mean total HCR-20 score was 19.25±5.26. The Historical, Clinical and Risk Management subscales showed mean scores of 8.33±2.96, 5.62±1.89, and 5.28±2.42, respectively.

The violent patients represented 45% of the sample.

The mean scores of the items H3, H10, C1, C2, C4 and R5 of HCR-20 were respectively : 1.33±0.79, 1.20±0.77, 1.22±0.88, 0.38±0.71, 1.30±0.64 and 1.28±0.73.

There was no statistical difference between the two groups in socio-demographic factors.

A history of suicidal attempts was significantly more common in the group of violent patients (p=0.029).

Regarding the HCR subscales, H3 score (relationship instability) and H10 score (Prior supervision failure) were significantly higher among violent patients (p=0.018 and 0.003 respectively). The C1 score (lack of insight), the C2 score (negative attitudes) and the C4 score (impulsivity) were also significantly higher among violent patients (p=0.016, 0.009 and 0.005 rescpectively).

The item R5 (stress) of the risk management subscale was significantly higher in the group of violent patients (p=0.003).

The total MOAS score detected severe agression in the nonviolent group (p=0.031).

**Conclusions:**

Our study suggests the efficacy of HCR-20 in identifying and distinguishing between violent and nonviolent patients with schizophrenia or schizoaffective disorder. The use of such reliable instrument in clinical psychiatric settings should be encouraged.

**Disclosure of Interest:**

None Declared

